# Weight Indices, Cognition, and Mental Health From Childhood to Early Adolescence

**DOI:** 10.1001/jamapediatrics.2024.1379

**Published:** 2024-06-03

**Authors:** Zhaolong Adrian Li, Mary Katherine Ray, Yueping Gu, Deanna M. Barch, Tamara Hershey

**Affiliations:** 1 Department of Psychiatry, Washington University School of Medicine in St Louis, Missouri; 2 Department of Psychological and Brain Sciences, Washington University in St Louis, Missouri

## Abstract

This cohort study evaluates the association between weight indices in childhood and changes in cognition and psychopathology.

High child and adolescent obesity rates (eg, 19.7% in the US) are problematic given links between early-life obesity and long-term health issues.^1^ While evidence suggests cross-sectional associations between obesity, lower cognitive functioning, and worse mental health in youth,^2^ it remains unclear whether these findings extend longitudinally and in what direction. Leveraging data from the Adolescent Brain Cognitive Development (ABCD) Study (release 5.0),^3,4^ we examined how weight indices for children aged 9 to 11 years are associated with changes in cognition and psychopathology across the 2 years thereafter, and vice versa.

## Methods

Baseline (June 2016 to October 2018) and 1- and 2-year follow-up ABCD Study data collected before COVID-19 (March 13, 2020) were included in this cohort study. Weight indices (body mass index [BMI]; waist circumference [WC]) and psychopathology were assessed annually^3^; cognition was assessed at baseline and 2 years^4^ (Table). Caregiver-reported race was assessed for reporting reasons. Caregivers and children provided written informed consent or assent to procedures approved by site institutional review boards. We followed the Strengthening the Reporting of Observational Studies in Epidemiology (STROBE) reporting guidelines.

**Table.** Participant Characteristics

Variable^a^	Particiapnts (N = 5269), mean (SD)^b^		
	Baseline	1-y Follow-up	2-y Follow-up
Age [range], y	10.0 (0.6) [8.9 to 11.1]	11.0 (0.6) [9.8 to 12.4]	11.9 (0.6) [10.6 to 13.7]
Sex, No. (%)			
Female	2522 (47.9)	NA	NA
Male	2747 (52.1)	NA	NA
Race and ethnicity, No. (%)^c^			
Asian	95 (1.8)	NA	NA
Black	511 (9.7)	NA	NA
Hispanic	955 (18.1)	NA	NA
White	3193 (60.6)	NA	NA
Other	515 (9.8)	NA	NA
Area deprivation index national percentile^d^	37.6 (25.5)	NA	NA
Income to needs ratio^e^	3.8 (2.4)	NA	NA
Pubertal development scale total score^f^	7.9 (2.3)	8.9 (2.9)	10.5 (3.5)
Body mass index^g^	18.4 (3.7)	19.4 (4.5)	20.3 (4.7)
WC, in (1 in = 2.54 cm)	26.2 (4.0)	27.5 (4.3)	28.5 (4.6)
NIHTB picture Vocabulary score	85.5 (7.9)	NA	89.7 (8.2)
NIHTB flanker Inhibitory Control score	95.0 (8.4)	NA	100.6 (7.1)
NIHTB pattern Comparison score	88.9 (14.4)	NA	103.8 (14.9)
NIHTB picture Sequence score	104.0 (12.1)	NA	110.2 (11.8)
NIHTB oral Reading Recognition score	91.6 (6.6)	NA	95.2 (6.4)
Little Man Task, No. correct	19.3 (5.5)	NA	24.0 (5.7)
RAVLT learning, No. correct	11.5 (2.4)	NA	11.4 (2.3)
RAVLT immediate recall, No. correct	9.9 (3.0)	NA	10.0 (2.6)
RAVLT delayed recall, No. correct	9.5 (3.1)	NA	9.4 (2.8)
CBCL total problems	16.7 (16.2)	16.1 (15.7)	15.3 (15.7)
CBCL internalizing behavior	4.8 (5.2)	4.8 (5.2)	4.7 (5.3)
CBCL externalizing behavior	4.0 (5.3)	3.7 (5.0)	3.6 (5.0)

Abbreviations: CBCL, Child Behavior Checklist; NA, not applicable; NIHTB, National Institutes of Health Toolbox; RAVLT, Rey Auditory Verbal Learning Test; WC, waist circumference.

^a^ Sociodemographic variables were assessed at baseline only. Cognition was assessed at baseline and 2-year follow-up using uncorrected scores that evaluated domains of verbal and reading ability (picture vocabulary, oral reading recognition), executive control and attention (flanker inhibitory control), processing speed (pattern comparison), learning and episodic memory (picture sequence, RAVLT), and visuospatial processing (Little Man Task).^4^ Higher scores reflect better performance. Annual psychopathology assessments using caregiver-reported CBCL raw scores included 11 empirical syndrome subscales (total problems [summarizing internalizing, externalizing, social, thought, and attention problems], internalizing [summarizing anxious/depressed, withdrawn/depressed, and somatic complaints], externalizing [summarizing rule-breaking and aggressive behavior], and 8 individual constituent subscales), 3 other subscales (sluggish cognitive tempo, obsessive-compulsive problems, and stress problems), and 6 subscales that were deemed cross-culturally consistent with *Diagnostic and Statistical Manual of Mental Disorders, Fifth Edition* categories (depression, anxiety, somatic, attention-deficit/hyperactivity disorder [ADHD], oppositional defiant, and conduct problems).^3^ For brevity, only characteristics of the total, internalizing, and externalizing problem subscales are shown here. Higher scores reflect greater endorsement.

^b^ For some variables, usable data may come from fewer participants due to missing data and/or excluded outliers (weight indices and cognition scores that were 4 SD or more away from the mean were removed). See the eMethods in Supplement 1 for details on participant data selection.

^c^ Response options were defined by the ABCD Study and included in the present study for descriptive but not analytical purposes. Other includes caregiver-reported American Indian or Alaska Native, Native Hawaiian or Other Pacific Islander, multiple races and/or ethnicities, and unknown race or ethnicity.

^d^ Based on American Community Survey (2011-2015) estimates at census block level. Higher percentiles reflect greater neighborhood socioeconomic disadvantage.

^e^ Calculated as the median level of household income bands divided by the 2017 US federal poverty guidelines given household size.

^f^ Based on caregiver-reported pubertal status ratings that previously showed high correlation with Tanner stages.^3^ Higher values reflect more advanced pubertal stage.

^g^ Body mass index is calculated as weight in kilograms divided by height in meters squared.

We used [age] × [baseline variable] interactions in linear mixed models to estimate associations between baseline BMI or WC and changes in cognition or psychopathology across time points, and vice versa. Models also included lower-order main effects, sociodemographic and developmental covariates, nested random intercepts, and random slopes (Figure). Sensitivity analyses explored (1) sex differences; (2) non-Gaussian distributions; and confounding of (3) weight-related medication (eg, psychostimulants) use, (4) common baseline psychiatric diagnoses, and (5) psychopathology covariates in baseline cognition models. We also ruled out any confounds from practice effects on the cognitive tasks. Significance was set at 2-sided P value of .05 corrected for false discovery rate (FDR). Data were analyzed from August 2023 to March 2024 using R software version 4.3.1 (R Project for Statistical Computing) (eMethods in Supplement 1).

**Figure.**
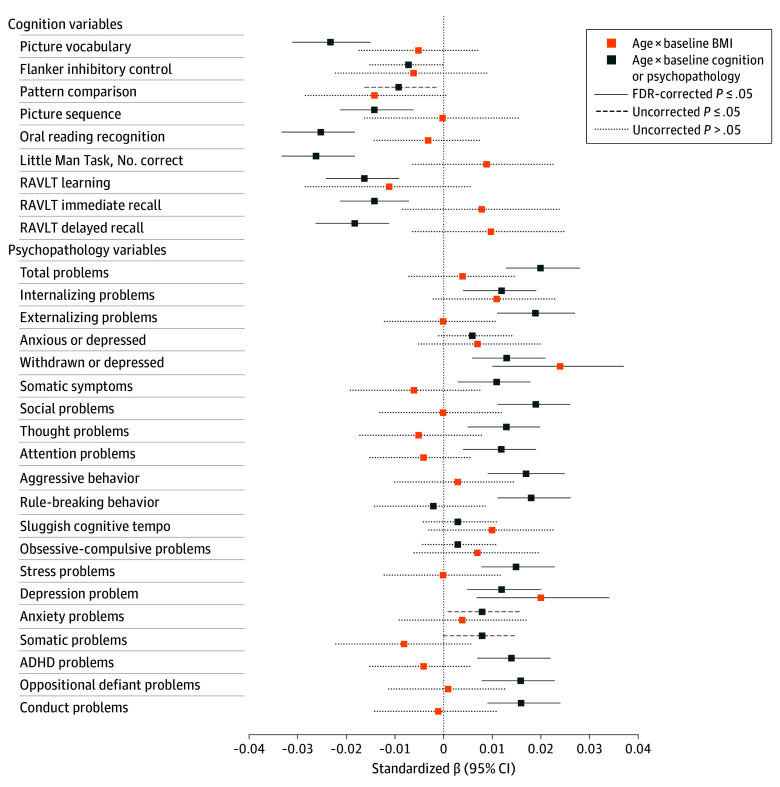
Associations Between Body Mass Index (BMI), Cognition, and Psychopathology as Children Enter Adolescence Standardized β coefficients with 95% profile likelihood CIs were longitudinal [age (centered at median baseline age of 10 y)] × [baseline BMI] interactions (orange) or [age] × [baseline cognition or psychopathology] interactions (blue), estimated in linear mixed models. Lower-order main effects were automatically added. Fixed-effect covariates included child sex, area deprivation index national percentile, income to needs ratio, pubertal development scale total score, and familial history of mental illness (depression, mania, psychosis, suicide attempt, and antisocial behavior) and drug or alcohol use problems. Random intercepts were participants nested within families within study sites. Participant-level random slopes were included, except for models where cognition was the outcome and assessed only twice. The same models were implemented when waist circumference (WC) was used as the weight index. Multiple comparison correction was performed within domains (cognition or psychopathology) and weight indices (BMI or WC) using false discovery rate at 2-tailed *P* ≤ .05. Full details on statistical modeling and sensitivity analyses that built on these models are shown in the eMethods in Supplement 1. ADHD indicates attention-deficit/hyperactivity disorder; FDR, false discovery rate; RAVLT, Rey Auditory Verbal Learning Test.

## Results

Characteristics of 5269 participants are shown in Table. Baseline BMI was not associated with longitudinal changes in cognition (Figure). Conversely, lower baseline cognition was overall associated with greater longitudinal BMI gain, including after adjusting for baseline psychopathology. In unstandardized estimates, for example, children who scored 1 point lower on baseline picture vocabulary had 0.012 (1.6%) more annual BMI (calculated as weight in kilograms divided by height in meters squared) gain than those scoring at median (IQR) of 85 (10).

Higher baseline BMI was associated with more longitudinal withdrawn or depressed symptoms and depression problems (Figure), with each 1 increase corresponding to 0.010 (22%) and 0.011 (15%) more problems annually beyond changes at median BMI. In equivalent clinical estimates, children with overweight or obesity (BMI ≥85th percentile) at baseline gained 0.07 (172%) and 0.06 (93%) more problems annually than those with normal weight (BMI ≥5th to <85th percentiles). Conversely, greater baseline psychopathology was broadly associated with greater BMI gain; eg, each baseline endorsement of externalizing problems corresponded to 0.015 (2.2%) more annual BMI increases compared with no endorsement.

Associations were not moderated by sex (FDR-corrected *P* ≥ .39). Results remained consistent in subgroups without weight-altering medications or common baseline psychiatric diagnoses and in non-Gaussian models. Findings with WC were similar to those with BMI, except baseline internalizing spectrum was not associated with longitudinal WC.

## Discussion

Lower cognitive performance and greater psychopathology at baseline were associated with increased weight gain as children entered adolescence, and higher baseline BMI was associated with more depressive symptoms over time. These longitudinal findings highlight the importance of cognitive and mental health to children’s healthy weight development and suggest that clinicians should monitor children with overweight or obesity for increased depression problems. Future work could explore plausible mediators of these associations, such as the role of caregiver-child conflicts and impaired reward learning or control in hampering adherence to healthy diet or lifestyle and thus exacerbating weight gain,^2,5,6^ and the role of body dissatisfaction-related stress and inflammation-mediated hypothalamic-pituitary-adrenal axis dysregulation in increased depression risk.^2,6^

Limitations include the lack of body composition measures and short longitudinal timeframe, which could have limited detection of later-emerging associations between weight and longitudinal cognition.^5^ Future ABCD Study data could allow us to assess the longer-term clinical value of our findings and identify biopsychosocial mediators.

## Notes

Supplement 1.
eMethods.
eReferences.

Supplement 2. Data Sharing Statement
