# Supplementary material for: Weight Indices, Cognition, and Mental Health From Childhood to Early Adolescence
Source: JAMA Pediatr. 2024 Jun 3;178(8):830–3. doi: 10.1001/jamapediatrics.2024.1379 (PMC11148784; doi:10.1001/jamapediatrics.2024.1379)
Supplement: Supplement 1. — eMethods. eReferences. [file jamapediatr-e241379-s001.pdf]

Supplemental Online Content

Li ZA, Ray MK, Gu Y, et al. Weight indices, cognition, and mental health from childhood to early adolescence.. *JAMA Pediatr*. Published online June 3, 2024.
doi:10.1001/jamapediatrics.2024.1379

eMethods.
eReferences.

This supplemental material has been provided by the authors to give readers additional information about their work.

© 2024 Li ZA et al. *JAMA Pediatrics.*

eMethods. Participant data selection; Main analyses (standardized estimates; unstandardized estimates); Sensitivity analyses (moderation by sex; no report of weight-related medication use; no report of common baseline psychiatric diagnoses; covarying for psychopathology in baseline cognition models; non-Gaussian models); Practice effects in cognitive scores

Participant data selection. Details on the Adolescent Brain Cognitive Development (ABCD) Study recruitment have been published elsewhere[1]. The ABCD Study is being conducted at 21 US sites and children were enrolled at baseline using a school-based recruitment system. The recruitment catchment of these sites was demographically representative of the US national and encompassed over 20% of US children aged 9-11 years. Children were included in the ABCD Study if they were 9-11 years old at baseline (June 2016 to October 2018) and fluent in English. Children who had history of severe neurological or psychiatric disorders, magnetic resonance imaging (MRI) contraindications, or were born prematurely (30 days) were excluded. The baseline ABCD cohort included 11864 children. In the present study, we additionally excluded children who had caregiver-reported history of cerebral palsy, brain tumor, brain aneurysm, brain hemorrhage, brain hematoma, stroke, epilepsy, seizures, traumatic brain injury, lead poisoning, multiple sclerosis, muscular dystrophy, intellectual disability, substance use disorder, schizophrenia, autism spectrum disorder, and other serious neurological or psychiatric conditions (n = 666). We further excluded children with diabetes (n = 81), eating disorders including anorexia nervosa, bulimia nervosa, and binge eating at present, in the past, or in remission (n = 241), or with casts/prostheses (n = 82). Lastly, given evidence of COVID-19-related weight gain in children[2] and disruptions in study administration, we included only participants whose baseline, 1-y, and 2-y data were all collected in-person before March 13, 2020 (date of US declaration of national emergency). A total of 6671 children met these inclusion and exclusion criteria. In all statistical analyses, we included certain covariates with missing data: area deprivation index national percentile (n = 333 missing); pubertal development scale total score (n = 46 missing); income-to-needs ratio (n = 572 missing); and familial history of mental illness (depression [n = 278 missing], mania [n = 261 missing], psychosis [n = 239 missing], suicide attempt [n = 264 missing], antisocial behavior [n = 151 missing]) and drug or alcohol use problems (n = 174 missing). Because listwise deletion was used in analyses, a maximum analytical sample of n = 5269 was eventually retained. See **Table** in main text for participant characteristics.

Main analyses (standardized estimates). For transparent data reporting and meta-analytical purposes, full results on standardized estimates of models presented in **Figure** in main text are available in a linked online data document at OSF https://tinyurl.com/Li-2024-BMICogPsych as eTable 1 (body mass index [BMI]) and eTable 2 (waist circumference [WC]).

Main analyses (unstandardized estimates). Full results on unstandardized estimates, referenced in main text, are available in eTable 3 (BMI) and eTable 4 (WC) in the linked online data document. These unstandardized models estimated changes in outcomes associated with per unit increase in baseline BMI, WC, cognition, or psychopathology

© 2024 Li ZA et al. *JAMA Pediatrics.*

(ie, [age] × [baseline variable] interactions) as well as age-related changes in outcomes (ie, main effect of [age]) at median baseline BMI, WC, or cognition, or zero baseline psychopathology endorsement. The ratio between the two (ie, proportion of change in trajectories) was also calculated. Accompanying Johnson-Neyman plots are shown in eFigures 1 and 2 in the linked online data document.

To further characterize the observed associations between baseline BMI and longitudinal psychopathology, we implemented additional models where participants were classified as having normal weight (age- and sex-adjusted BMI percentiles ≥ 5th to < 85th) or overweight/obesity (BMI percentiles ≥ 85th) at baseline per the Centers for Disease Control and Prevention 2000 growth charts[3]. We assessed associations between this binary weight category variable and longitudinal psychopathology. Full results are reported in eTable 5 in the linked online data document.

Sensitivity analyses (moderation by sex). Main models were modified such that the [age] × [baseline variable] interactions were extended to [age] × [baseline variable] × [sex] interactions in order to assess if the longitudinal associations were significantly different between sexes. Full results are available in eTable 6 (BMI) and eTable 7 (WC) in the linked online data document.

Sensitivity analyses (no report of weight-related medication use). As weight-changing medications could confound the association between weight indices and psychopathology, we reran the main models in subgroups of participants who did not have caregiver-reported weight-related medication use in the two weeks prior to each study visit. Based on Verhaegen et al.[4], we screened for use of ***antidepressants*** (amitriptyline [Elavil®], nortriptyline [Pamelor®, Aventyl®], imipramine [Tofranil®], desipramine [Norpramin®], doxepin [Sinequan®, Silenor®], clomipramine [Anafranil®], escitalopram [Lexapro®], paroxetine [Paxil®, Pexeva®, Brisdelle®], citalopram [Celexa®], fluoxetine [Prozac®, Rapiflux®, Sarafem®, Selfemra®], sertraline [Zoloft®], duloxetine [Cymbalta®, Drizalma Sprinkle®, Irenka®], venlafaxine [Effexor®], phenelzine [Nardil®], buproprion [Aplenzin®, Budeprion®, Buproban®, Forfivo®, Wellbutrin®, Zyban®], trazodone [Desyrel®, Oleptro®], nefazodone [Serzone®], mirtazapine [Remeron®], maprotiline [Ludiomil®]), *antipsychotics* (molindone [Moban®], haloperidol [Haldol®], perphenazine [Etrafon®], aripiprazole [Abilify®], ziprasidone [Geodon®], lurasidone [Latuda®], paliperidone [Invega®], iloperidone [Fanapt®], asenapine [Saphris®], amisulpride [Barhemsys®], quetiapine [Seroquel®], risperidone [Risperdal®], clozapine [Clozaril®, FazaClo®, Versacloz®], olanzepine [Zyprexa®]), *mood stabilizers* (lithium [Eskalith®, Lithobid®]), *anticonvulsants* (topiramate [Eprontia®, Qudexy®, Topamax®, Topiragen®, Trokendi®], zonisamide [Zonegran®], lamotrigine [Lamictal®], levetiracetam [Elepsia®, Keppra®], tiagabine [Gabitril®], clonazepam [Klonopin®], oxcarbazepine [Trileptal®], gabapentin [FusePaq Fanatrex®, Gabarone®, Gralise®, Neurontin®], pregabalin [Lyrica®], valproic acid/ divalproex sodium [Depakene®, Depakote®, Stavzor®], carbamazepine [Carbatrol®, Epitol®, Equetro®, Tegretol®]), *attention-deficit/hyperactivity disorder (ADHD) medications* (methylphenidate [Aptensio®, Concerta®, Cotempla®, Jornay®, Metadate®, Methylin®, QuilliChew®, Quillivant®,

© 2024 Li ZA et al. *JAMA Pediatrics.*

Ritalin®], dextroamphetamine [Dexedrine®, Dextrostat®, Liquadd®, ProCentra®, Zenzedi®], dexmethylphenidate [Focalin®], amphetamine [Adzenys®, Dyanavel®, Evekeo®], lisdexamfetamine [Vyvanse®], combined [Adderall®], atomoxetine [Strattera®], guanfacine [Intuniv®, Tenex®]), *growth hormones*, *thyroid hormones* (levothyroxine [Levothroid®, Levoxyl®, Synthroid®, Tirosint®, Unithroid®]), and *diabetes medications* (insulin, metformin [Fortamet®, Glucophage®, Glumetza®, Riomet®]). Full results are available in eTable 8 (BMI) and eTable 9 (WC) in the linked online data document.

Sensitivity analyses (no report of common baseline psychiatric diagnoses). We reran the main models in subgroups of participants who did not have caregiver-reported history of ADHD, depression, bipolar disorder, anxiety, or phobias diagnoses at baseline. Full results are available in eTable 10 (BMI) and eTable 11 (WC) at the website above.

Sensitivity analyses (covarying for psychopathology in baseline cognition models). To assess if psychopathology confounded the observed associations between baseline cognition and longitudinal BMI and WC, we extended these models by adding each of the 20 baseline psychopathology variables as covariate, running a total of 180 models each for BMI and WC. Full results are available in eTable 12 in the linked online data document

Sensitivity analyses (non-Gaussian models). To address the possible concern that psychopathology outcomes could follow non-Gaussian distributions, we reran the main models where psychopathology outcomes were fit using Poisson, zero-inflated Poisson, negative binomial, and zero-inflated negative binomial distributions. The best model for each psychopathology outcome was selected based on convergence, Akaike information criterion, Bayesian information criterion, and log-likelihood. Results from these models are consistent with those from the main models and are available in eTable 13 in the linked online data document.

Practice effects in cognitive scores. Repeated cognitive testing may yield experience-driven performance improvement that confounds with changes due to brain and cognitive development. Although the ABCD Study cognitive assessment frequency (every other year) and tasks were designed to minimize practice effects[5], Anokhin et al. had reported significant practice effects seen at 2-y follow-up relative to baseline[6]. Here, we replicated their analyses by comparing cognitive performance in age-matched pairs where one participant datapoint came from baseline (first assessment) and the other from 2-y follow-up (second assessment). A total of 1550 children were in the age overlap of 10.6 y (127 mo) to 11.1 y (133 mo) across the two timepoints (**eMethods Figure**, panel **A**), of which 561 pairs had matching age, sex, race and ethnicity, area deprivation index national percentile, income-to-needs ratio, baseline age- and sex-adjusted BMI and WC z-scores, and baseline pubertal development scale total score established using 1-to-1 Mahalanobis distance matching (**eMethods Figure**, panel **B**).

© 2024 Li ZA et al. *JAMA Pediatrics.*

We estimated practice effects in cognitive scores by subtracting the baseline data from the 2-y follow-up data in each matched pair, finding very similar values to the estimates reported by Anokhin et al. (**eMethods Table 1**)[6]. These estimates were potentially generalizable to our entire study sample (n = 5269), because the 2-y follow-up participants (who demonstrated practice effects) had similar characteristics compared to all included participants except in terms of income-to-needs ratio. Fisher's exact test or Wilcoxon rank sum test showed: sex (*P* > .99), race and ethnicity (*P* = .32), area deprivation index national percentile (*P* = .29), income-to-needs ratio (*P* = .004), baseline BMI z-score (*P* = .09), baseline WC z-score (*P* = .57).

eMethods Figure.

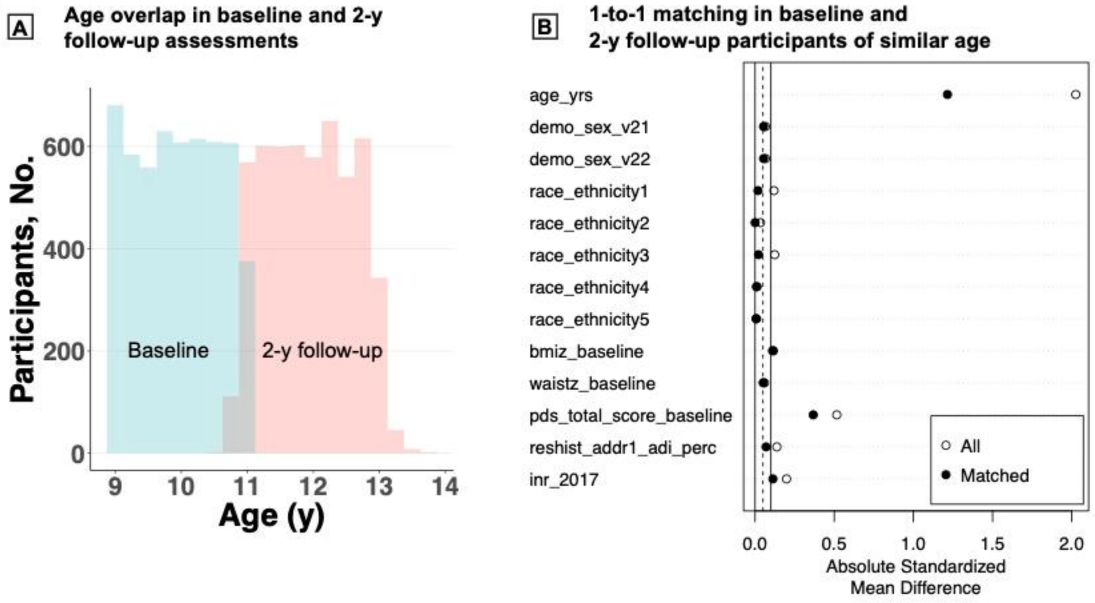

Caption. Note the age difference shown in panel **B** among matched pairs was small (0.12 y, or 1.4 mo) even though there was a noticeable absolute standardized mean difference.

eMethods Table 1. Estimated practice effects (n = 561)

**Variable**	**Mean (SD)**	**Median**	**Anokhin et al.**[6]
NIHTB Picture Vocabulary score	-0.71 (9.75)	-1	-0.84
NIHTB Flanker Inhibitory Control score	1.24 (10.27)	0	1.94
NIHTB Pattern Comparison score	4.33 (19.42)	4	5.87
NIHTB Picture Sequence score	2.17 (16.74)	2	2.93
NIHTB Oral Reading Recognition score	0.27 (8.45)	0	-0.43
Little Man Task, n correct	1.33 (7.76)	1	1.97
RAVLT learning, n correct	-0.65 (3.14)	-1	-0.75
RAVLT immediate recall, n correct	-0.60 (3.73)	-1	-0.84
RAVLT delayed recall, n correct	-0.76 (3.92)	-1	-0.89

© 2024 Li ZA et al. *JAMA Pediatrics.*

Importantly, our practice effect estimates were mostly uncorrelated with baseline BMI or WC (**eMethods Table 2**); ie, children gained the same amount of improvement from repeated assessments irrespective of their baseline weight status, consistent with a prior finding[7]. Therefore, any association between baseline weight indices and longitudinal changes in cognition would likely be driven by age-related developmental changes and not practice effects.

eMethods Table 2. Pearson correlations between baseline weight and practice effects

	Baseline BMI		Baseline WC	
**Estimated practice effects**	***r***	***P* value**	***r***	***P* value**
NIHTB Picture Vocabulary score	-0.05	.25	0.00	.94
NIHTB Flanker Inhibitory Control score	-0.08	.05	-0.03	.47
NIHTB Pattern Comparison score	0.00	.98	0.01	.90
NIHTB Picture Sequence score	-0.02	.65	-0.01	.75
NIHTB Oral Reading Recognition score	-0.05	.22	0.01	.87
Little Man Task, n correct	0.01	.75	0.05	.23
RAVLT learning, n correct	-0.03	.55	0.01	.79
RAVLT immediate recall, n correct	0.05	.23	0.10	.01
RAVLT delayed recall, n correct	0.04	.33	0.09	.04

© 2024 Li ZA et al. *JAMA Pediatrics.*

eReferences

1. Garavan H, Bartsch H, Conway K, et al. Recruiting the ABCD sample: Design considerations and procedures. *Dev Cogn Neurosci*. 2018;32:16-22. doi:10.1016/j.dcn.2018.04.004
2. Betts SS, Adise S, Hayati Rezvan P, et al. Socioeconomic Adversity and Weight Gain During the COVID-19 Pandemic. *JAMA Pediatr*. 2023;177(10):1102-1105. doi:10.1001/jamapediatrics.2023.2823
3. Kuczmarski RJ, Ogden CL, Guo SS, et al. 2000 CDC Growth Charts for the United States: methods and development. *Vital Health Stat 11*. 2002;(246):1-190.
4. Verhaegen AA, Van Gaal LF. Drugs That Affect Body Weight, Body Fat Distribution, and Metabolism. In: Feingold KR, Anawalt B, Blackman MR, et al., eds. South Dartmouth (MA); 2000.
5. Luciana M, Bjork JM, Nagel BJ, et al. Adolescent neurocognitive development and impacts of substance use: Overview of the adolescent brain cognitive development (ABCD) baseline neurocognition battery. *Dev Cogn Neurosci*. 2018;32:67-79. doi:10.1016/j.dcn.2018.02.006
6. Anokhin AP, Luciana M, Banich M, et al. Age-related changes and longitudinal stability of individual differences in ABCD Neurocognition measures. *Dev Cogn Neurosci*. 2022;54:101078. doi:https://doi.org/10.1016/j.dcn.2022.101078
7. Sullivan E V, Brumback T, Tapert SF, et al. Effects of prior testing lasting a full year in NCANDA adolescents: Contributions from age, sex, socioeconomic status, ethnicity, site, family history of alcohol or drug abuse, and baseline performance. *Dev Cogn Neurosci*. 2017;24:72-83. doi:https://doi.org/10.1016/j.dcn.2017.01.003

© 2024 Li ZA et al. *JAMA Pediatrics.*
