# Supplementary material for: Weight Indices, Cognition, and Mental Health From Childhood to Early Adolescence
Source: JAMA Pediatr. 2024 Jun 3;178(8):830–3. doi: 10.1001/jamapediatrics.2024.1379 (PMC11148784; doi:10.1001/jamapediatrics.2024.1379)
Supplement: Supplement 2. — Data Sharing Statement [file jamapediatr-e241379-s002.pdf]

Data Sharing Statement

Li. Weight Indices, Cognition, and Mental Health From Childhood to Early Adolescence. *JAMA Pediatr*. Published June 03, 2024. doi:10.1001/jamapediatrics.2024.1379

Data
Data available: Yes
Data types: Deidentified participant data, Data dictionary
How to access data: Data in this study were from the ABCD Release 5.0 (accessed September 26, 2023; https://doi.org/10.15154/8873-zj65). The ABCD Study repository grows and may be modified as new data are collected and processed. A data dictionary is available as part of the Release Notes at https://wiki.abcdstudy.org/release-notes/start-page.html.
When available: beginning date: 06-16-2023

Supporting Documents
Document types: Other (please specify)
Additional Information: For transparent data reporting and meta-analytical purposes, full data results are available in a Linked Online Data Document, referenced throughout the Supplement.
How to access documents: The Linked Online Data Document is available at https://tinyurl.com/Li-2024-BMICogPsych.
When available: beginning date: 03-12-2024

Additional Information
Who can access the data: Access to the ABCD Study data is open only to researchers with a valid National Institute of Mental Health Data Archive (NDA) Data Use Certification (DUC). Access to the Linked Online Data Document is open to everyone.
Types of analyses: Pediatric research purposes.
Mechanisms of data availability: The ABCD Study data is available upon approval of National Institute of Mental Health Data Archive (NDA) Data Use Certification (DUC). The Linked Online Data Document is openly available.
